# The Performance of the Two-Seeded GdBCO Superconductor Bulk with the Buffer by the Modified TSMG Method

**DOI:** 10.3390/mi14050987

**Published:** 2023-04-30

**Authors:** Yufeng Zhang, Chunyan Li, Ziwei Lou, Penghe Zhang, Yan Zhang, Shuangyuan Shen, Guanjie Ruan, Jiaying Zhang

**Affiliations:** 1 College of Mathematics and Physics, Shanghai University of Electric Power, Shanghai 201306, China; 2 Shanghai Key Laboratory of High Temperature Superconductors, Shanghai University, Shanghai 200444, China

**Keywords:** GdBCO superconductor bulk, buffer layer, two-seeded technology, critical current density

## Abstract

The multiseeding technique is a method to grow large-sized REBa_2_Cu_3_O_7−*δ*_ (REBCO, where *RE* is a rare earth element) high temperature superconducting bulks. However, due to the existence of grain boundaries between seed crystals, the superconducting properties of bulks are not always better than those of single grain bulks. In order to improve the superconducting properties caused by grain boundaries, we introduced buffer layers with a diameter of 6 mm in the growth of GdBCO bulks. Using the modified top-seeded melt texture growth method (TSMG), that is, YBa_2_Cu_3_O_7−*δ*_ (Y123) as the liquid phase source, two GdBCO superconducting bulks with buffer layers with a diameter of 25 mm and a thickness of 12 mm were successfully prepared. The seed crystal arrangement of two GdBCO bulks with a distance of 12 mm were (100/100) and (110/110), respectively. The trapped field of the GdBCO superconductor bulks exhibited two peaks. The maximum peaks of superconductor bulk S_A_ (100/100) were 0.30 T and 0.23 T, and the maximum peaks of superconductor bulk S_B_ (110/110) were 0.35 T and 0.29 T. The critical transition temperature remained between 94 K and 96 K, with superior superconducting properties. The maximum *J*_C, self-field_ of S_A_ appeared in specimen b5, which was 4.5 × 10^4^ A/cm^2^. Compared with S_A_, the *J*_C_ value of S_B_ had obvious advantages in a low magnetic field, medium magnetic field and high magnetic field. The maximum *J*_C, self-field_ value appeared in specimen b2, which was 4.65 × 10^4^ A/cm^2^. At the same time, it showed an obvious second peak effect, which was attributed to Gd/Ba substitution. Liquid phase source Y123 increased the concentration of the Gd solute dissolved from Gd211 particles, reduced the size of Gd211 particles and optimized *J*_C_. For S_A_ and S_B_ under the joint action of the buffer and the Y123 liquid source, except for the contribution of Gd211 particles to be the magnetic flux pinning center with the improvement of *J*_C_, the pores also played a positive role in improving the local *J*_C_. More residual melts and impurity phases were observed in S_A_ than in S_B_, which had a negative impact on the superconducting properties. Thus, S_B_ exhibited a better trapped field and *J*_C_.

## 1. Introduction

There have been decades of research history since the discovery of (RE)Ba_2_Cu_3_O_7−_*_δ_* ((RE)BCO, where *RE* is a rare earth element) high-temperature superconductors. RE(BCO) high-temperature superconductors have broad application prospects and huge application potential in high-tech fields, such as superconducting maglev trains, flywheel energy storage systems, and superconducting bearings [1,2,3,4,5]. The development of RE(BCO) high-temperature superconductors is gradually becoming mature, but in its research process, there are also many problems that need to be solved. The single-grain high-temperature superconductor bulks manufactured by the top-seeded melt growth process (TSMG) have been studied [6,7,8] and are usually grown as parallelepiped with (100), (010), and (001) crystal planes [9]. However, the superconducting performance is unable to reach the expected results because of its slow growth rate and microstructure defects. Traditional TSMG technology has many defects due to the outflow of the liquid phase in the heating process, the generation of oxygen, and unstable factors in the powder pressing process, such as the obvious shrinkage of the final sample, the existence of a large number of pores, and the region without the second phase particles in the microstructure [10,11,12]. In order to solve these problems, many new research methods have been proposed sequentially. Infiltration growth (IG) methods can supply enough liquid sources to reduce the shrinkage of the final bulks, compared with the TSMG method [13]. The bulks grown by Melt-Powder-Melt-Growth (MPMG) have fewer weak links, bigger superconductor grains, smaller RE211 particles, and less pores [14,15].

Buffer-assisted technology is a recognized effective method to improve the performance of high-temperature superconductor bulks [16,17,18,19,20]. By optimizing the composition and the aspect ratio of the buffer layer, Namburi et al. [21] improved the problems related to interfacial stress and seed contamination, and then YBCO bulk superconductors with a diameter of 25~35 mm were successfully prepared. As recorded in Ref. [20], first, the use of the buffer greatly improves the seeding success rate, which can prevent mutual pollution between the seed crystal and precursor block and can absorb the macro cracks caused by lattice mismatch so as to improve the superconducting properties and thermal stability of the seed crystal. Second, the buffer can be used as a large secondary seed to induce the homogeneous epitaxial growth of the main particles. Increasing the size of the c-grown region and the crystallinity of the grown crystal can also homogenize the distribution of the Gd211 particles and improve the critical current density. With the simultaneous introduction of the buffer and the rich liquid phase, GdBCO-Ag bulk samples with a diameter of 48 mm have been successfully prepared [22].

Multiseeding technology is expected to prepare large-size REBCO bulk samples [23,24,25,26]. However, multiseeding technology will reduce the critical current density characteristics of superconductor bulks due to the existence of a residual impurity phase at the grain boundary between seed crystals. Therefore, only when the grain boundaries are eliminated are large overcurrent loops generated, resulting in large magnetic moments and trap magnetic fields [21]. Samples with seed crystals placed in (110/110) mode have relatively clean grain boundaries [27]. In addition, Shi et al. used bridge seeding technology to achieve the best alignment in the preparation of multi-seeded samples. They found that the seed (110/110) arrangement has more advantages than the seed (100/100) arrangement because this arrangement can form clean grain boundaries [28,29]. As documented in reference [30], samples with different arrangements have different growth patterns at grain boundaries. If two grains impinging each other are planar parallel (100/100), each grain grows in the opposite direction and then meets and stops at the grain boundary, where the residual melt cannot continue. On the other hand, if two grains impacting each other are not parallel in plane (110/110), and the grains meet and continue to grow along the grain boundary, there will be no residual melt.

Compared with other light rare earth elements, the GdBCO superconducting bulk has the most stable performance and the lowest Gd/Ba substitution effect, and the element substitution to a lesser extent can deepen the second peak effect and improve the critical current density. In this paper, the superconducting properties and microstructure of GdBCO high temperature superconductor bulks prepared by the combination of two- seeded technology in different arrangement and buffer technology are studied, which is a new test to grow the high performance of the superconductor bulks. The superconductor material prepared in this paper is a GdBCO bulk. Double NdBCO seeds and double buffer GdBCO superconductor bulks with a diameter of 25 mm were prepared by the modified top-seeded melt growth process (TSMG), and the YBa_2_Cu_3_O_7−_*_δ_* (Y123) pellet was introduced as the liquid phase source to avoid the shrinkage problem caused by liquid phase loss [31,32]. The effects of different arrangement of seed crystals on the properties of the bulks were studied. The superconducting properties were analyzed by the trapped field, critical current density (*J*_C_), critical transition temperature (*T*_C_) and microstructure.

## 2. Experimental

According to GdBa_2_Cu_3_O_7−*δ*_ + 40 mol% Gd_2_BaCuO_5_ + 10 wt%Ag_2_O + 0.5 wt%Pt, commercial GdBa_2_Cu_3_O_7−*δ*_ (Gd123, 99.9%) powder was mixed with Gd_2_BaCuO_5_ (Gd211, 99.9%) powder in the molar ratio of 1:0.4, and 10 wt% Ag_2_O and 0.5 wt% Pt were added to improve the mechanical strength and refine Gd211 particles. They were ground evenly with a mortar and then pressed into a cylindrical pellet with a diameter of 25 mm and a thickness of 12 mm as the precursor. Commercial Y123 (99.9%) powder was used in this experiment as a liquid phase source; it was pressed into a cylindrical pellet with a diameter of 25 mm and a thickness of 3 mm. The chemical compositions of buffer pellets were similar to those of precursors, except for not adding Ag_2_O and Pt powder. After being fully ground, they were pressed into cylindrical pellets with a diameter of 6 mm and a thickness of 3 mm. Seed crystals with a size of 2 × 2 × 0.5 mm^3^ NdBCO were used to guide the growth.

**Figure 1:**
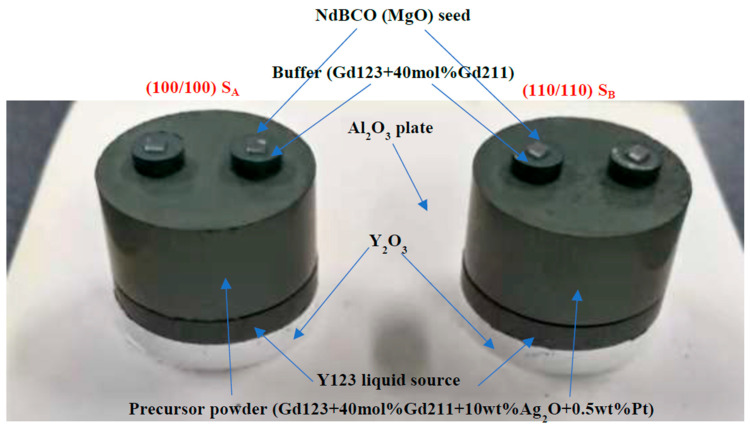
Schematic assembly of pellets.

The pressed pellets were assembled in order from top to bottom: NdBCO seed crystals, buffers, precursor, Y123 liquid source, Y_2_O_3_ and aluminum oxide plate. A Y_2_O_3_ cylindrical pellet with equal diameter was inserted between the Al_2_O_3_ plate and the Y123 pellet to prevent a reaction between them. As shown in Figure 1, two NdBCO seed crystals were arranged in two different ways: (100/100), and (110/110), named S_A_ and S_B_. The distance between the two seed crystals (the linear distance between the center of the seed crystal) was 12 mm.

**Figure 2:**
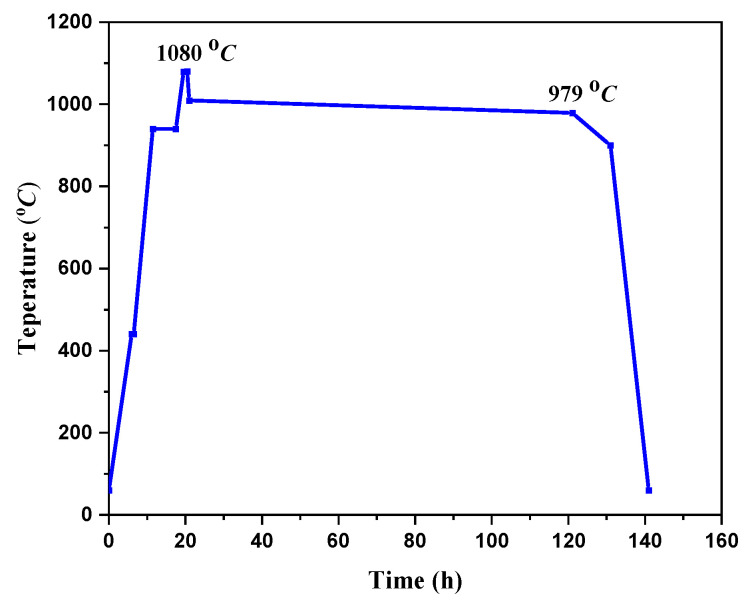
The temperature curve of the superconductor bulk S_A_ and S_B_.

The assembled pellet was placed into a box furnace for sintering, and the temperature was raised to 1080 °C at a rate of 50 °C/h, held for 1 h, quickly cooled to 1009 °C, and then slowly cooled to 979 °C at a rate of 0.3 °C/h. Finally, it was cooled to room temperature with the furnace, as shown in Figure 2. In order to obtain the orthogonal superconducting phase structure, annealing treatment was carried out in a flowing high-purity oxygen atmosphere. S_A_ and S_B_ were heated to 450 °C within 5 h, kept for 40 h, and then cooled to 350 °C within 40 h and 300 °C within 30 h, respectively. In the end, they were naturally cooled to room temperature. Then, two GdBCO bulks with high performance were fabricated successfully.

**Figure 3:**
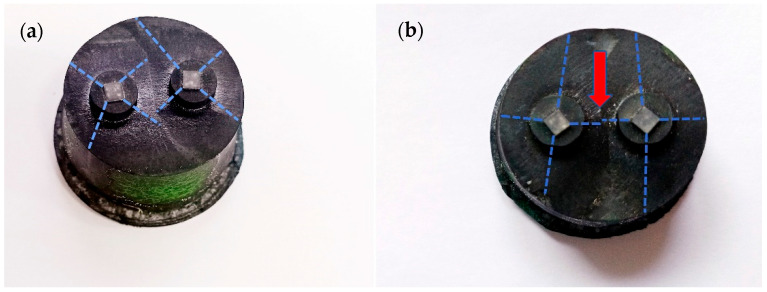

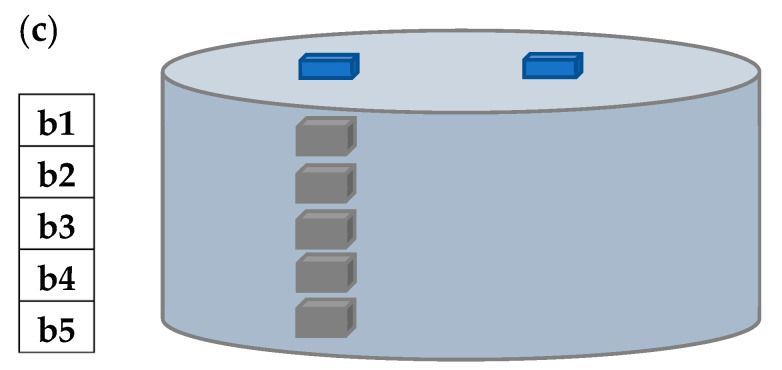
Macromorphology of S_A_ (**a**) and S_B_ (**b**). The blue dotted line indicates the growth facet line on the top surface of the sample, and the red arrow indicates a slight drift between the two seed crystals. (**c**) Schematic diagram of the positions of two specimens cut from the GdBCO superconductor bulk.

The top and bottom surfaces of the bulks were polished to measure the trapped field. Under an external magnetic field of 1.5 T and liquid nitrogen environment for 30 min, the external magnetic field was removed, and the trapped magnetic field curve was tested by a Hall probe sensor with a distance of 2 mm from the surface of the bulks. In order to study the effect of the buffer layer on the performance of the bulk, based on the growth patterns of two bulk materials, namely along the direction parallel to the edge of the seed crystal, we cut five specimens of b1, b2, b3, b4 and b5 from S_A_ and S_B_ under the position of the seed crystal. The surface of the bulks was polished about 1 mm, and the b1~b5 specimens with a size of 2 mm × 2 mm × 1 mm were took from the upper surface to the bottom, as shown in Figure 3c. The physical property measurement system (PPMS) was used to measure the DC magnetization of specimens cut at different positions of the bulks. The critical current density (*J*_C_) was calculated according to the extended Bean critical state model. The microstructure of the bulks was observed by scanning electron microscopy (SEM).

## 3. Results and Discussion

The macromorphology of S_A_ and S_B_ is shown in Figure 3. The growth facet line of the seed crystal is marked with a blue dotted line in the picture. It can be seen that the seeds were intact, and there was no melting phenomenon. There was an obvious drift phenomenon between the two seed crystals of S_A_, and the two seed crystals of S_B_ were arranged relatively orderly, but there was still a slight drift phenomenon (indicated by the red arrow). From the top views, we can clearly see the grain boundary. The appearance of grain boundaries is inevitable in the growth process, so we need to reduce the negative impact of grain boundaries on the superconductor bulk. Many factors affect the grain boundary. Theoretically, the factors affecting grain boundary depth are the distance between seed crystals. The arrangement of the seed crystals and the buffer layer and the size of the buffer layer are also important factors when using the buffer layer. The depth of the grain boundary increases with the increase of the distance between seed crystals for the growth of the multiseeded bulk. The depth of the grain boundary of multiseeded bulks is mainly determined by the angle [33] formed by the top surface and the boundary of the a and c growth region. The way to change this angle is to increase or reduce the distance between the seed crystals. The larger the angle, the deeper the grain boundary is formed, indicating that the distance between the seed crystals is farther. The smaller the angle, the shallower the grain boundary formed, indicating that the distance between the seed crystals is closer. The seed crystal and the buffer layer can effectively induce growth as a large-size seed when the buffer layer is added, reduce the effective length between the seed crystals significantly, reduce the a-sector growth area and increase the c-sector growth area. Then, the angle formed between the top surface and the boundary of the a-sector and c-sector growth area is reduced so as to decrease the depth of the grain boundary, which improves the superconducting properties [17,34,35,36].

**Figure 4:**
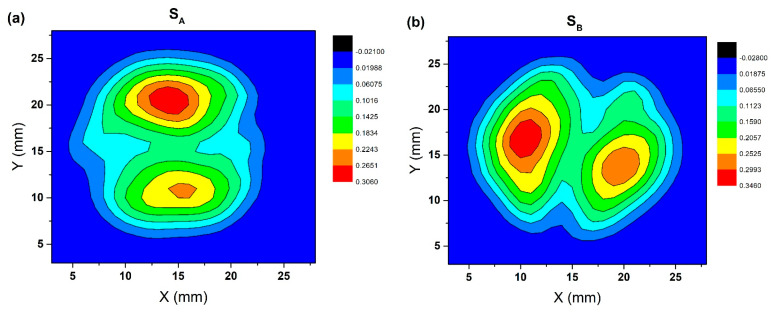

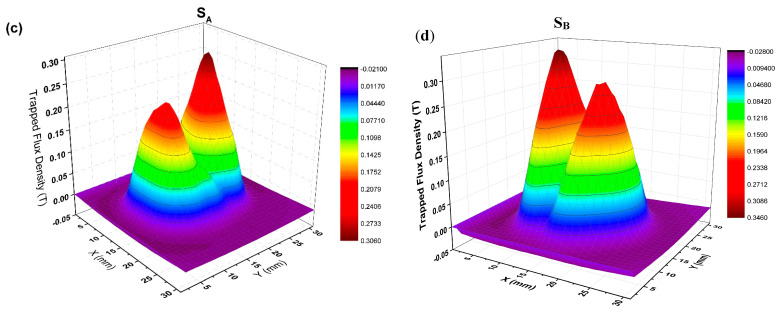
The top surface trapped field (3D map and 2D map) of S_A_ and S_B_ measured in a liquid nitrogen atmosphere, (**a**) 2D map of S_A_; (**b**) 2D map of S_B_; (**c**) 3D map of S_A_; (**d**) 3D map of S_B_, respectively.

Figure 4 shows the distribution of trapped magnetic flux density on the top surface of two bulks measured in a liquid nitrogen environment (3D map and 2D map). It can be seen from the picture that the magnetic flux distribution of the two bulks is conical and shows two peaks. The two peaks of S_A_ were 0.30 T and 0.23 T, and the two peaks of S_B_ were 0.35 T and 0.29 T, respectively. S_B_ had a smaller difference between the two peaks and showed a higher peak compared with S_A_. It can be observed in (a) and (b) of Figure 4 that the two growth regions were not symmetrical, and the nucleation particles guided by one seed crystal were significantly better than the other. This phenomenon may be caused by the different nucleation times of two seed crystals, the different temperatures at the different positions of the seed crystals, or the asymmetric arrangement of two-seeded crystals, which should occur randomly [28].

The symmetry of the arrangement of two-seeded crystals of S_A_ (100/100) was not high, which led to poor connectivity in the grains and wrong crystal orientation between the growth regions to form polluted grain boundaries. The accumulation of non-superconducting phases is the obstruction of the flow of the superconducting current in the current circuit, which leads to the poor performance of the trapped field [20,37,38]. In contrast, the seed crystal arrangement symmetry of S_B_ was higher. In the (110/110) seed crystal arrangement, the grains met at the grain boundary along the growth direction, which continued to grow to form a relatively clean grain boundary. Therefore, both the value of the peak and the difference between the two peaks were better, which suggests that the trapped field of S_B_ was better than S_A_. Therefore, we infer that the trapped field distribution of the GdBCO superconductor bulk guided by a multiseed crystal is highly related to the symmetry of the seed crystal and the existence of an impurity phase at the boundary. The higher the symmetry of the seed crystal, the cleaner the grain boundary, and the better the trapped field performance.

**Figure 5:**
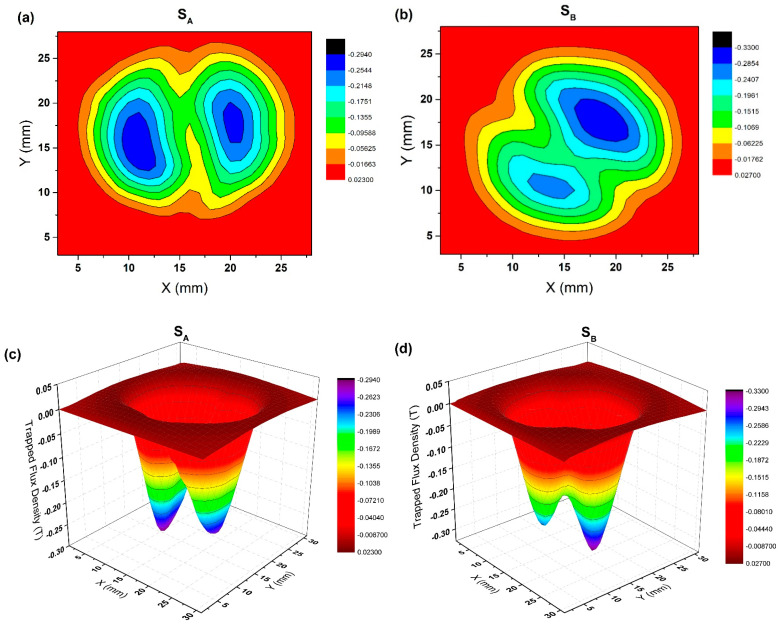
Bottom surface trapped field (3D map and 2D map) of S_A_ and S_B_ measured in liquid nitrogen atmosphere, (**a**) 2D map of S_A_; (**b**) 2D map of S_B_; (**c**) 3D map of S_A_; (**d**) 3D map of S_B_, respectively.

Figure 5 shows the distribution of trapped magnetic flux density on the bottom surface of GdBCO superconductor bulks measured in a liquid nitrogen environment (3D map and 2D map). The magnetic flux distribution of the two bulks was conical and showed two peaks. We also found that the two peaks of S_A_ were 0.29 T and 0.27 T, and the two peaks of S_B_ were 0.29 T and 0.33 T. Compared with the top surface, the difference between the two peaks of each bulk on the bottom surface was smaller. This should be a positive effect brought about by the introduction of Y123 as a liquid source in the experiment, which has a significant effect on improving the uniformity of trapped field distribution [39]. Inserting the Y123 phase into the bottom of the precursor not only successfully solves the problem of liquid phase loss in the growth process but also accumulates a sufficient liquid phase at the growth front and plays a great role in leading to the more uninform of the distribution of Gd211 particles in the Gd123 matrix. As we all know, the distribution of Gd211 particles in the Gd123 matrix is an important index to judge the performance of the bulks because they are not only an important source of the flux pinning center, but they also reduce the residue of the unreacted liquid phase to the greatest extent. In addition, the introduction of the Y123 liquid phase source also expands the growth region of c orientation and inhibits the formation of a high angle grain boundary, which plays a positive role in the formation of an excellent trapped field [40].

**Figure 6:**
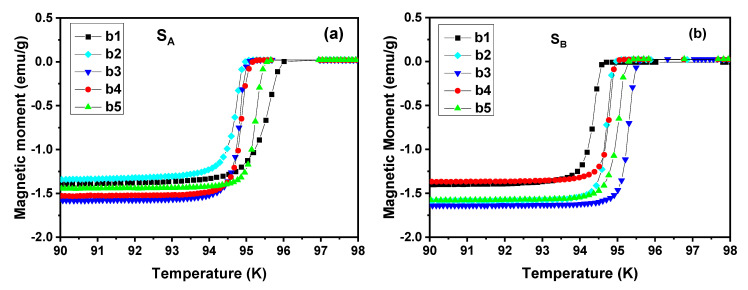
Temperature dependence of magnetization of specimens cut below the seed crystals of S_A_ (**a**) and S_B_ (**b**), respectively.

Figure 6 shows the temperature dependence of the magnetization of specimens b1, b2, b3, b4 and b5 cut below the seed crystals of S_A_ and S_B_. The curves in Figure 6a,b show that the values of initial transition temperature *T*_C, onset_ is between 94~96 K with the superior superconducting properties. On the whole, the transition width of S_A_ changes greatly, which is not as sharp as S_B_. The distribution of *T*_C, onset_ of S_A_ was relatively concentrated, showing a large transition width at position b1, while it was relatively narrow at the other positions. The distribution of *T*_C, onset_ of S_B_ was relatively scattered, but the transition width of all specimens was very sharp except b1, and the value of transition width was about 0.5 K. Among them, the *T*_C, onset_ of position b1 was the lowest, and the *T*_C, onset_ of position b3 was the highest. This suggests that the transition width of *T*_C_ in specimen b1 near the seed crystals slightly increases due to the substitution of Gd/Ba ions [25,31]. We infer that the addition of two independent buffer layers has no adverse effect on the superconducting properties of GdBCO bulks.

**Figure 7:**
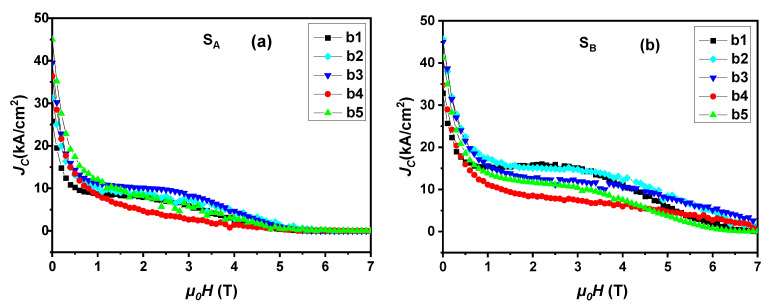
The critical current density curve measured by cutting the specimens below the seed crystal of S_A_ (**a**) and S_B_ (**b**), respectively.

**Figure 8:**
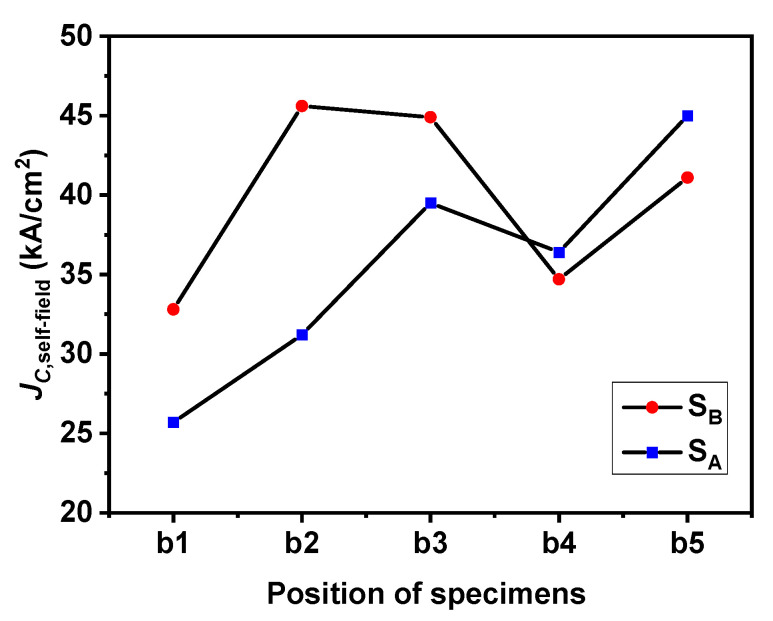
Plots of the *J*_C_ in self-field of specimens from different positions under seed crystal.

Figure 7 shows the critical current density curve of specimens b1, b2, b3, b4 and b5 under S_A_ and S_B_ seed crystals. For convenience in the analysis, the self-field *J*_C_ values of the specimens at different positions are plotted in Figure 8. The *J*_C_ curve results of S_A_ showed that under the applied magnetic field, the *J*_C, self-field_ value of the specimens had different trends. Specimen b5 at the bottom had the highest *J*_C, self-field_ under the low field and was lower than b3 under the middle field. Its highest *J*_C, self-field_ value was 4.5 × 10^4^ A/cm^2^. The *J*_C_ curve results of S_B_ showed that under the applied magnetic field, the *J*_C, self-field_ of the specimens changed with the increase in the distance from the seed crystal in the c-axis direction, increased from position b1 to b3, and was higher than the value of S_A_ at the corresponding position, but the values of positions b4 and b5 were slightly lower than that of S_A_. The maximum *J*_C, self-field_ value of specimen b2 was 4.56 × 10^4^ A/cm^2^, which is comparable to the results of GdBCO superconductor bulks [25,29]. S_B_ maintained absolute advantages in low magnetic field, medium magnetic field and high magnetic field. The *J*_C_ value of the bottom specimens of the bulks was very high, which may have been due to the introduction of a Y123- rich liquid phase, which plays a positive role in filling the defects in the microstructure completely and improving the uniformity of superconducting phase particle distribution [39], consistent with the results in trapped fields.

The applied magnetic field started from 5 T; the *J*_C_ value of the curve in S_A_ was almost zero, while S_B_ was much better. Under low magnetic field and medium magnetic field, S_B_ showed a higher *J*_C, self-field_ value and more neat curve trend. Moreover, there was a second peak effect near the seed crystal. This effect shows the superiority in the performance of the trapped field. The *T*_C_ at the corresponding position decreased, and this effect decreased with the increase in the distance from the seed crystal, which is attributed to Gd/Ba substitution [41,42]. The additional Y123 liquid source increases the concentration of Gd solute decomposed from Gd211 particles to strengthen the Gd/Ba substitution effect and improve *J*_C_. Because there is a temperature gradient in the process of bulk growth, especially at a higher temperature near the seed crystal, the Gd/Ba substitution effect is more serious, which shows the most obvious second peak phenomenon [43].

**Figure 9:**
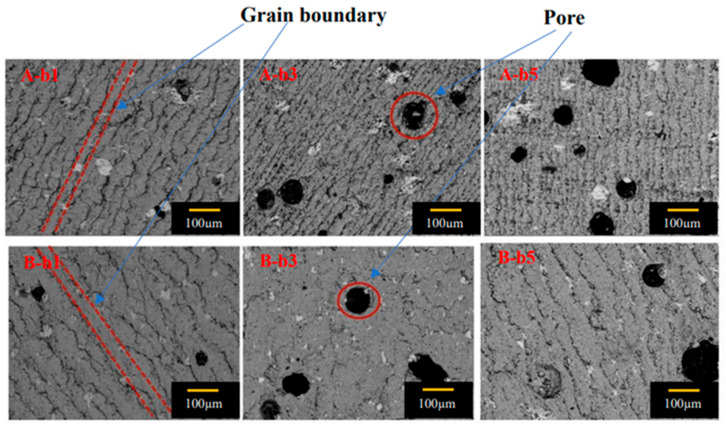
Scanning electron microscope photos of 500 times magnification of cut specimens of S_A_ (100/100) and S_B_ (110/110) at positions b1, b3 and b5.

Figure 9 is a set of scanning electron microscope (SEM) photographs of specimens b1, b3 and b5 of S_A_ (100/100) and S_B_ (110/110) at 500 times magnification. The pore distribution in the pictures showed typical characteristics, which changed with the distance from the seed crystal. The existence of these pores was mainly due to the precipitation of the gas in the melting process, which were trapped in the melt and forms circular pores. The distribution trend of the pores of two bulks in b1, b3 and b5 was consistent. The number and size of pores at b1 were small, and the number and size of pores at b3 and b5 were large. Compared with that in S_A_, the grain boundary was narrow and the amount of residual melt at the grain boundary was small in S_B_ specimens, which was related to the high trapped field in S_B_, as shown in Figure 4 and Figure 5. The pore distribution of S_A_ showed that the number of pores in the bottom was higher than that in the middle, and the middle was higher than that in the top. The pore distribution of S_B_ showed that the number in the middle was higher than that in the bottom, and the number in the bottom was higher than that in the top position. Overall, the number of pores in S_A_ was significantly higher than that in S_B_.

**Figure 10:**
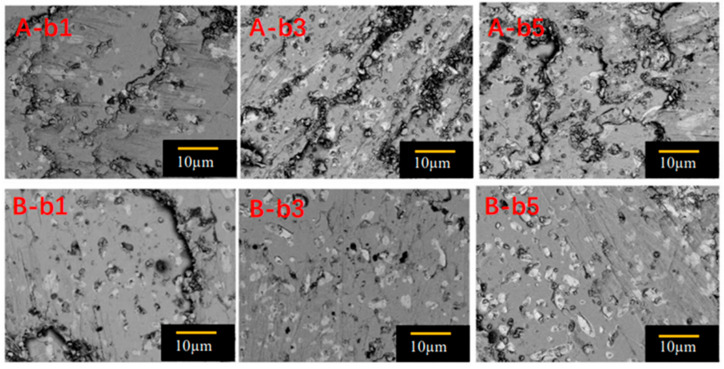
Scanning electron microscope photos of 5000 times magnification of cut specimens of S_A_ (100/100) and S_B_ (110/110) at positions b1, b3 and b5.

**Figure 11:**
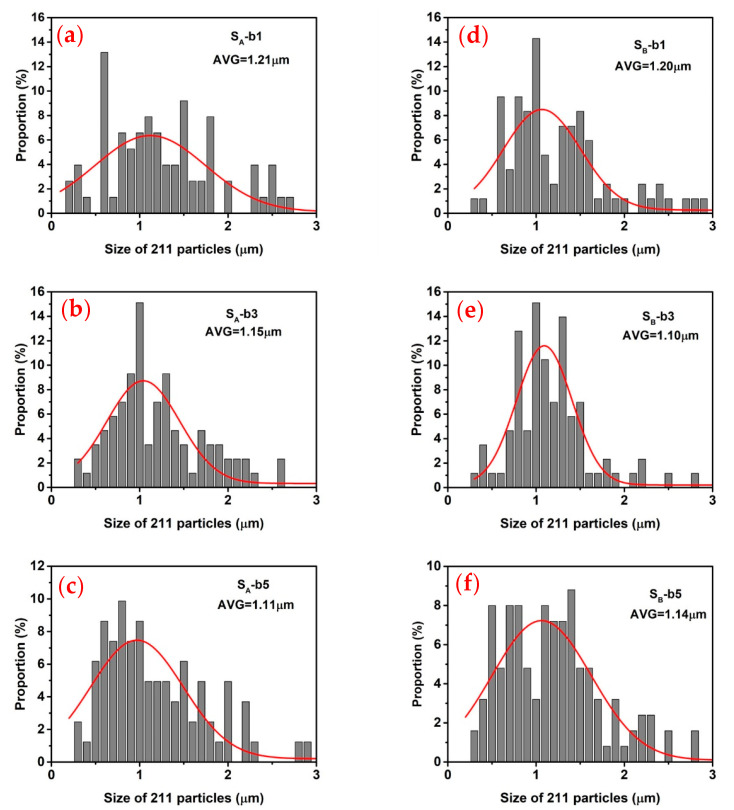
The size distribution of the Gd211 particles in the GdBCO superconductor bulks at positions b1, b3 and b5. The red solid curves represent the fitted curves obtained by fitting the size distribution of the Gd211 particles. The average size values of the Gd211 particles (AVG) obtained from the peak position of each fitted curve are also given, (**a**) S_A_-b_1_; (**b**) S_A_-b_3_; (**c**) S_A_-b_5_; (**d**) S_B_-b_1_; (**e**) S_B_-b_3_; (**f**) S_B_-b_5_, respectively.

Figure 10 shows scanning electron microscope photographs of specimens b1, b3 and b5 of S_A_ (100/100) and S_B_ (110/110) at 5000 times magnification. In order to further analyze the Gd211 particles distributed in these GdBCO bulks, the size of the Gd211 particles shown in Figure 10 was determined by the software of Nano measure 1.2, as shown in Figure 11. The average size values of the Gd211 particles (AVG) were obtained from the peak position of each fitted curve. The size of Gd211 particles near the seed crystal was the largest and changed with the increase in the distance from the seed crystal. As shown by comparing the average size of Gd211 particles, the average value of b3 belonging to S_B_ was the smallest, followed by b5 of S_A_, which had a maximum *J*_C_, as shown in Figure 7. This shows that the smaller the size of Gd211 particles, the larger the *J*_C_ value. Generally, a low-concentration Gd211 region will be formed near the seed crystal. However, in the SEM image of b1, a large number of evenly distributed Gd211 particles was observed, which is a positive effect brought about by the use of a buffer layer [16]. According to the pushing/trapping theory [44], the number of Gd211 distributed at different positions increases with the increase of the distance, and the effect of the particle refinement is better. Due to the addition of the Y123-rich liquid phase, the uniformity of the second phase particles is improved to increase the superconducting performance at this position. From the microstructure (SEM) observation of the bulks, the small change in the volume fraction of Gd211 particles along the c-axis is not effective in explaining the change trend of *J*_C_. Then, there are more pores in the area with a high *J*_C_ value, which suggests that pores of a suitable size may form an effective flux pinning center to improve the local *J*_C_. For S_A_ and S_B_ under the joint action of buffer and Y123 liquid source, except for the contribution of Gd211 particles to be the magnetic flux pinning center with the improvement of the *J*_C_, porosity also affects performance. Figure 7 shows that the *J*_C_ of b1 was the lowest, while b3 and b5 increased significantly. For S_A_, b5 > b3 > b1, and for S_B_, b3 > b5 > b1. The pore distribution of these specimens was consistent with the change trend of *J*_C_, and the pores played a positive role in improving the superconducting properties.

The microstructure observation of S_A_ showed that they were in the solidified liquid phase, and there were impurities that hindered the flow of the superconducting current and reduced the superconducting performance [45]. Moreover, there were many large-sized Gd211 particles that could not be used as an effective flux pinning center to reduce *J*_C_. The microstructure observation results of S_B_ showed that the solidified liquid phase and impurities were significantly less than that of S_A_. It was clean and flat under a scanning electron microscope (SEM), indicating that S_B_ possessed higher and superior superconducting performance, as demonstrated by the exhibition of the trapped field and *J*_C_.

## 4. Conclusions

The buffer layer with a diameter of 6 mm and YBa_2_Cu_3_O_7−_*_δ_* (Y123) liquid source were introduced to successfully prepare two GdBCO superconductor bulks with two NdBCO seed crystal arrangements of (100/100) and (110/110) in the distance of 12 mm and then their superconducting properties were carefully analyzed. The diameter and thickness of the precursor of two GdBCO superconductor bulks were 25 mm and 12 mm, respectively. The maximum peaks of both GdBCO superconductor bulks appeared on the top surface, which were 0.30 T in S_A_ and 0.35 T in S_B_. The use of the buffer layer reduced the depth of the grain boundary and improved the trapped field properties of bulks as a whole. The difference in the peak values of the two bulks was related to the arrangement of seed crystals. The low peak value of S_A_ was due to the existence of a residual melt and impurity phase at the grain boundary, which hindered the movement of superconducting current and led to a poor trapped field. The grain boundary of S_B_ was relatively clean, so its trapped field was better. The magnetic flux distribution on the bottom surface showed similar and high characteristics due to the introduction of the rich Y123 liquid phase. The rich Y123 liquid phase can promote the uniform distribution of Gd_2_BaCuO_5_ (Gd211) particles, expand the growth region of c orientation and inhibit the formation of high angle grain boundaries. The critical transition temperature of the bulks was 94–96 K, with good superconducting properties. In the *J*_C_~μ_0_H curve, S_B_ had obvious advantages in a low magnetic field, medium magnetic field and high magnetic field. An obvious second peak effect was observed in the specimens near the seed crystal position, which made the *J*_C_ value increase and the *T*_C_ at the corresponding position decrease, and this effect decreased with the increase in the distance from the seed crystal position due to the effect of Gd/Ba substitution. Moreover, the Y123 liquid source increased the concentration of Gd solute dissolved from Gd211 particles and strengthened the Gd/Ba substitution effect. Based on the distribution of Gd211 and pores in superconductor bulks, the smaller the size of the Gd211 particles, the higher the *J*_C_ of the bulk. The distribution of pores was consistent with the trend of the *J*_C_. This suggests that pores also have a positive effect on the superconducting properties of the superconductor bulks. More residual melts and impurity phases existed in S_A_ with the two-seeded arrangement of (100/100), resulting in slightly lower superconducting properties compared with that of S_B_ with the two-seeded arrangement of (110/110). The experimental data will be important for the preparation of large-sized superconductor bulks.
